# Thermal Deformation Behavior and Microstructural Evolution of Multicomponent Mg-Li-Zn-Al-Y Alloys under Hot Compression

**DOI:** 10.3390/ma17020489

**Published:** 2024-01-19

**Authors:** Kun Yang, Weiwu Bai, Bin Li, Hao Chen, Guo Li, Guobing Wei, Junwei Liu

**Affiliations:** 1 International Joint Laboratory for Light Alloys (MOE), Chongqing University, Chongqing 400044, China; yangk51203@163.com (K.Y.); cailiaoren_bww@163.com (W.B.); mkakaroto@163.com (B.L.); c13996093337@163.com (H.C.); liguo_edu@163.com (G.L.); 2 National Key Laboratory of Advanced Casting Technologies, Chongqing University, Chongqing 400044, China; 3 School of Industrial Engineering, Ningxia Polytechnic, Ningxia 750021, China; 4 School of Materials Science and Engineering, Zhengzhou University of Aeronautics, Zhengzhou 450046, China

**Keywords:** multicomponent alloy, microstructural evolution, constitutive equation, processing map

## Abstract

High-temperature compression tests on Mg-11.5Li-2.5Zn-0.35Al-0.3Y (in wt.%) were carried out on a Gleeble-3500 thermal simulator. Flow stress and microstructural evolution were analyzed at different temperatures (T = 473 K, 523 K, 573 K, and 623 K) and strain rates ($\dot{\varepsilon }$ = 1 s^−1^, 0.1 s^−1^, 0.01 s^−1^, and 0.001 s^−1^). On this basis, the constitutive model of the alloy was established using the Arrhenius-type constitutive model, and the thermal processing map of the alloy was drawn based on the DMM (dynamic material modeling) theory. The experimental results show that the flow stress of the Mg-11.5Li-2.5Zn-0.35Al-0.3Y alloy decreases with an increase in temperature and a decrease in strain rate. The grain size increases uniformly with the increase in temperature, while a sudden increase occurs with the decrease in strain rate. The predicted value of the model is compared with the experimental value to verify the correctness of the model, and the correlation coefficient, R = 0.9690, was calculated, which further proves the applicability of the model to the Mg-11.5Li-2.5Zn-0.35Al-0.3Y alloy. This alloy can be safely plastic-deformed 473 K~623 K and 0.001 s^−1^~1 s^−1^.

## 1. Introduction

In recent years, magnesium alloys have attracted widespread attention due to their excellent properties, such as their high strength-to-weight ratio, high specific stiffness, good thermal conductivity, and low density (1.3~1.9 g/cm^3^; density: 2/3 of aluminum alloys and 1/4 of steel) [1,2,3]. These properties make magnesium alloys have a broad development prospect for the automotive, aerospace, military, 3C (computer, communication, and consumer electronics), and electronics industries [4,5,6,7]. However, due to the HCP structure of magnesium alloys and limited openable slip system at room temperature, they lead to some disadvantages, such as poor plasticity at room temperature, which greatly limits the widespread application of magnesium alloys [8]. Therefore, many current studies have focused on the high-temperature deformation of magnesium alloys to improve their plasticity by activating more slip systems, so as to overcome the disadvantage of the poor plasticity of magnesium alloys [9]. Some people have also overcome this disadvantage by alloying, with Li being deemed a better choice. Li is the lightest metal material (0.543 g/cm^3^); adding Li to Mg can not only obtain ultra-light Mg-Li alloys (1.35~1.65 g/cm^3^) but also can reduce the axial ratio (c/a) of Mg, which leads to the reduction in the critical shear stress (CRSS) level of the slip system, and thus more slip systems are activated [10,11,12]. At the same time, according to the Mg-Li binary phase diagram, when the Li content is higher than 5.7 wt.%, the HCP structure of Mg begins to change to the BCC structure. When the Li content is higher than 10.3 wt.%, the HCP structure of Mg will absolutely change to the BCC structure; thus, it can overcome the disadvantages of magnesium alloys with poor plasticity to a large extent [10]. But at the same time, the strength of Mg-Li alloys will also be reduced. Zn and Al have high solid solubility in Mg, which can improve the strength of Mg-Li alloys through solid solution strengthening and second-phase strengthening. Zhao et al. [13] added Al and Li to Mg-Y-Zn alloys; the alloy’s microstructure was obviously more homogeneous and finer, accompanied by the precipitation of the Al-Li phase and the nanometal Al_0.9_Li_34.3_Mg_64.5_Zn_0.3_ phase; the tensile strength increased from 278 MPa to 299 MPa; and the elongation increased from 1% to 14.1%. Moreover, adding rare-earth elements (REs) can produce precipitation strengthening and fine grain strengthening effects, which have a significant effect on the strength of Mg-Li alloys. Wu et al. [14] added Y to Mg-8Li-Al and Mg-8Li-3Al, which refined and spheroidized the alloy’s microstructure; moreover, their alloy formed the Al_2_Y phase that exists at the grain boundaries, the elongation of the alloy increased from 40% to 60%, and the ultimate strength increased from 117 MPa to 140 MPa. Therefore, in this study, it was meaningful to study the hot deformation behavior of a multicomponent Mg-Li-Zn-Al-Y system alloy. At present, many studies have been carried out on the hot deformed behavior and the constitutive relationship of Mg-Li alloys, but there are few studies on Mg-Li alloys with the BCC structure in this aspect.

At present, on the one hand, the research on BCC-structured Mg-Li alloys mainly focuses on corrosion resistance and mechanical properties, while the research on the microstructural evolution process is rarely implemented yet. On the other hand, the amount of research on BCC-structured multifunctional Mg-Li alloys is much less than that on HCP- structured and HCP + BCC-structured Mg-Li alloys, and the exploitation of this alloy is still insufficient. Meanwhile, the presence of many elements will produce a variety of complex phases, which have a more complex effect on microstructural evolution. Therefore, it is necessary to study the microstructural evolution of BCC-structured Mg-Li alloys, which can provide a certain reference for the subsequent research on the hot deformation mechanism of BCC-structured Mg-Li alloys. At present, the more widely used thermal deformation characterization method is the Arrhenius-type constitutive model, which has been proven to be helpful for the study of high-temperature plastic deformation of materials. B. Venkatesh et al. [15] and Yang et al. [16] have already used this characterization method to study the high-temperature plastic deformation of Mg-2.08Ag-2.07Nd-0.6Zr and Mg-9Li-3Al-2.5Sr alloys, respectively. Among them, the Zener–Hollomon hyperbolic sine equation ($\dot{\varepsilon }$ = *A*[*sinh*(*ασ*)]*^n^exp*[−*Q*/*RT*]) links the deformation temperature, strain rate, and flow stress through mathematical relationships, which is of great significance for the accuracy of numerical simulations and optimization of the process parameters. Xu et al. [17] and Wu et al. [18] imported the calculated constitutive models of the Mg-6Gd-5Y-0.3Zr and AZ61 alloys into finite element software ABAQUS for simulation, and the results showed that finite element analysis combined with the constitutive relations reliably predicted the thermal deformation behavior of these alloys.

The purpose of this study was that thermal compression experiments on the Mg-11.5Li-2.5Zn-0.35Al-0.3Y alloy will be carried out using the Gleeble-3500 thermal simulation test system; the stress–strain curves of the alloys will be plotted; the constitutive equations will be calculated; and the thermal processing maps will be drawn using the experimental data of thermal compression. The correctness of the thermal processing maps was verified by analyzing the microstructure of the deformed alloy.

## 2. Materials and Methods

In this study, the alloys were prepared by melting high-purity Mg, Li, Zn, and Al (>99.9% (wt.%)) materials, and Y was added to the alloys in the form of a Mg-10Y intermediate alloy. The materials were cleaned and polished to remove surface grease and oxide layers before melting. To prevent the evaporation and oxidation of Mg and Li, melting was carried out in a resistance furnace at 973.15 K under an argon atmosphere. The melt was homogeneously stirred and then poured into a graphite die and cooled to room temperature in air to form a Mg-11.5Li-2.5Zn-0.35Al-0.3Y alloy cast billet with a final geometry of Φ80 mm × 150 mm. An inductively coupled plasma emission spectrometer (ICP-AES, Agilent 5110(OES), Santa Clara, CA, USA) was used to determine the chemical composition of the samples, and the chemical composition of the samples was Mg-11.5Li-2.5Zn-0.35Al-0.3Y.

Subsequently, a homogeneous treatment was carried out in a vacuum furnace, set at a temperature of 573 K, for 4 h. Then, a uniaxial hot compression specimen with geometrical dimensions of Φ10 mm × 15 mm was taken from a cross-section of the ingot using a wire cutter. A series of thermal compression experiments were then carried out on a Gleeble-3500 thermal simulator at the temperatures of 473 K, 523 K, 573 K, and 623 K. The strain rates were 0.001 s^−1^, 0.01 s^−1^, 0.1 s^−1^, and 1 s^−1^, and were stopped when the strain reached 60%. These cylindrical specimens were heated to a predetermined temperature at a heating rate of 10 K/s and held for 20 s to ensure the uniformity of the specimen microstructure, and the specimens were quenched immediately after the compression procedure was completed to retain the original microstructure of the specimen’s microstructure.

X-ray diffraction (XRD, D/Max-2500PC, Tokyo, Japan) experiments were performed on the thermally compressed specimens to determine the phases contained in the Mg-11.5Li-2.5Zn-0.35Al-0.3Y specimens. Then, the compressed specimens were cut out along the central axis into trapezoidal arcs with a height of 10 mm, an upper base of 4 mm, and a lower base of 6 mm, polished, and corroded, and the corrosion solution was chosen to be a 4% alcoholic nitrate solution. Optical microscope (OM, OLYMPUS PMG3, Shinjuku, Japan), scanning electron microscopy (SEM, FEI NOVA NANOSEM 400, Brno, Czech Republic), and energy-dispersive spectroscopy (EDS) analyses were carried out in order to study the microstructures. Based on the OM plots, the grain size was counted using Image-Pro Plus 6.0, and then OriginPro 2021 was used to fit the grain size distribution using the peak fitting method.

## 3. Results and Discussion

### 3.1. The Microstructure of the Alloys in a Hot Compression State

**Figure 1:**
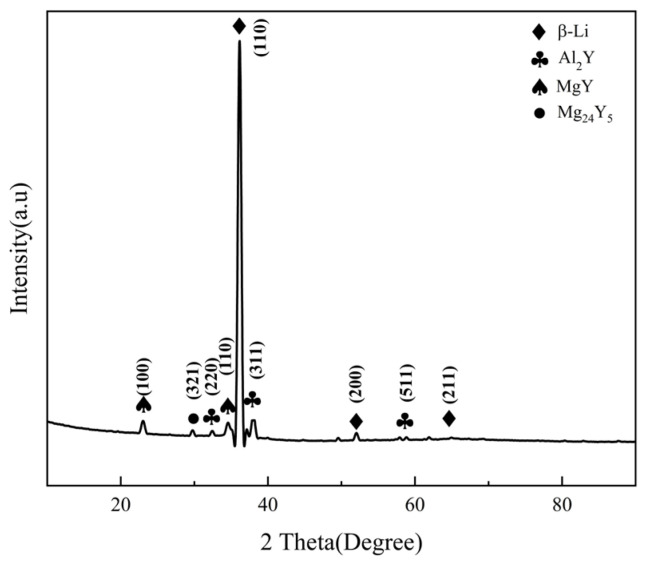
XRD pattern of the as-cast Mg-11.5Li-2.5Zn-0.35Al-0.3Y alloy.

The phase composition of the Mg-11.5Li-2.5Zn-0.35Al-0.3Y alloy was determined through XRD mapping analysis (Figure 1). This alloy has a principal phase composition comprising a β-Li phase (a matrix phase; a solid solution of Mg solidly dissolved in Li), an Al_2_Y phase (a precipitated phase; a solid phase with a FCC structure), a MgY phase (a precipitated phase; a solid phase with a cubic crystal structure), and a Mg_24_Y_5_ phase (a precipitated phase; a solid phase with a FCC structure).

**Figure 2:**
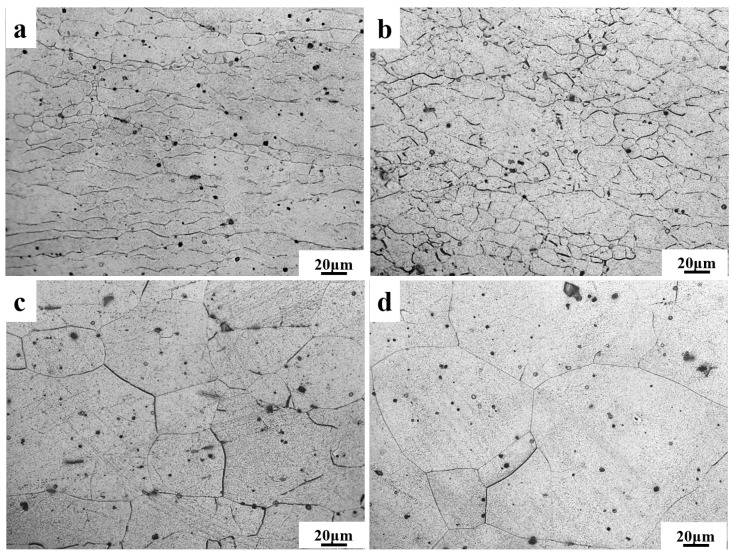
Microstructure of the Mg-11.5Li-2.5Zn-0.35Al-0.3Y alloy at T = 623 K and *ε* = 0.6 with different strain rates: (**a**) 1 s^−1^, (**b**) 0.1 s^−1^, (**c**) 0.01 s^−1^, and (**d**) 0.001 s^−1^.

**Figure 3:**
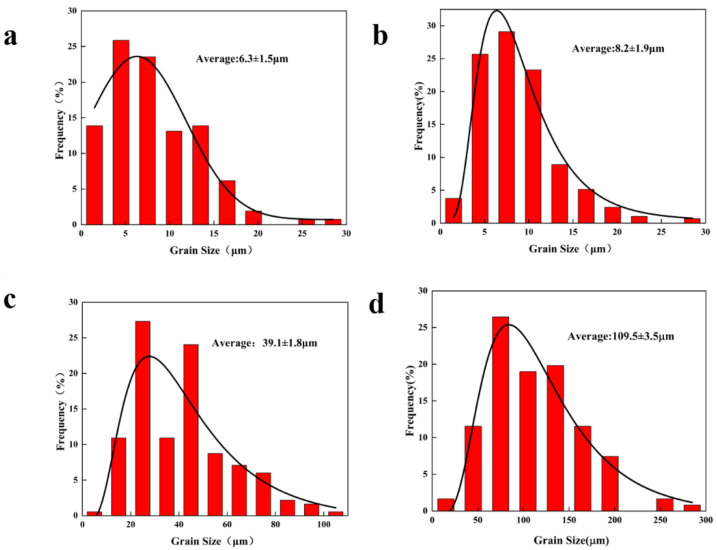
Grain size plots of the Mg-11.5Li-2.5Zn-0.35Al-0.3Y alloy at T = 623 K and *ε* = 0.6 with different strain rates: (**a**) 1 s^−1^, (**b**) 0.1 s^−1^, (**c**) 0.01 s^−1^, and (**d**) 0.001 s^−1^.

The microstructure and corresponding grain size of the Mg-11.5Li-2.5Zn-0.35Al-0.3Y alloy at different strain rates are shown in Figure 2 and Figure 3, respectively. It can be observed that with the decrease in the strain rate, the shape and size of the grains undergo a significant change. A large number of elongated deformed grains are presented in Figure 2a, while the grains in Figure 2b–d are dominated by equiaxed grains. This is mainly because of the fact that when the strain rate is 1 s^−1^, although the 623 K already meets the dynamic recrystallization temperature, most of the deformed grains are retained due to the extremely short deformation time. As the strain rate decreases, the degree of dynamic recrystallization of deformed grains increases. The average grain size increases with the decreasing strain rate, and the average grain size increases from 8.2 ± 1.9 μm (Figure 3b) to 39.1 ± 1.8 μm (Figure 3c) with the decreasing strain rate from 0.1 s^−1^ to 0.01 s^−1^, which is about 4.7 times. With the decrease in the strain rate from 0.01 s^−1^ to 0.001 s^−1^, the average grain size increases from 39.1 ± 1.8 μm (Figure 3c) to 109.5 ± 3.5 μm (Figure 3d), an increase of about 2.8 times. The variation trend in grain size generally matches the variation trend in stress. Combined with the subsequent thermal processing maps at 623 K, this is sufficient for plastic deformation processing at high strain rates ($\dot{\varepsilon }$ = 1 s^−1^).

**Figure 4:**
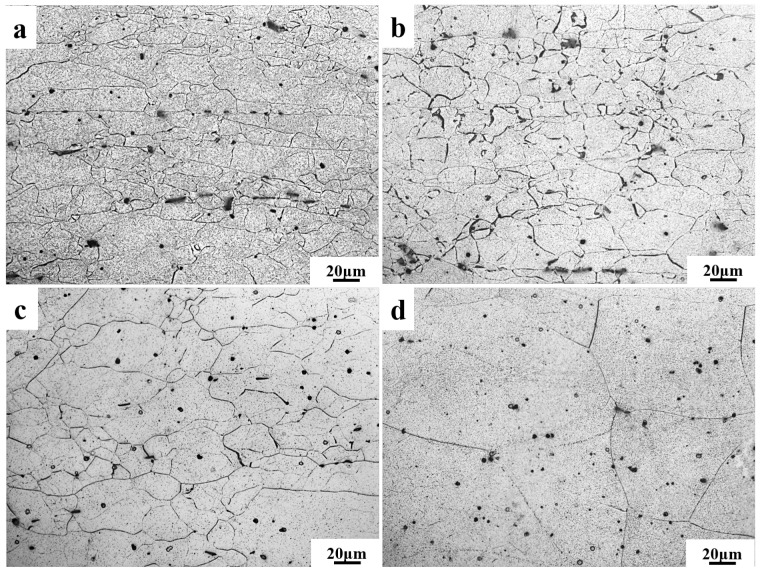
Microstructures of the Mg-11.5Li-2.5Zn-0.35Al-0.3Y alloy at $\dot{\varepsilon }$ = 0.001 s^−1^ and *ε* = 0.6 with different temperatures at 0.001 s^−1^: (**a**) 473 K, (**b**) 523 K, (**c**) 573 K, and (**d**) 623 K.

**Figure 5:**
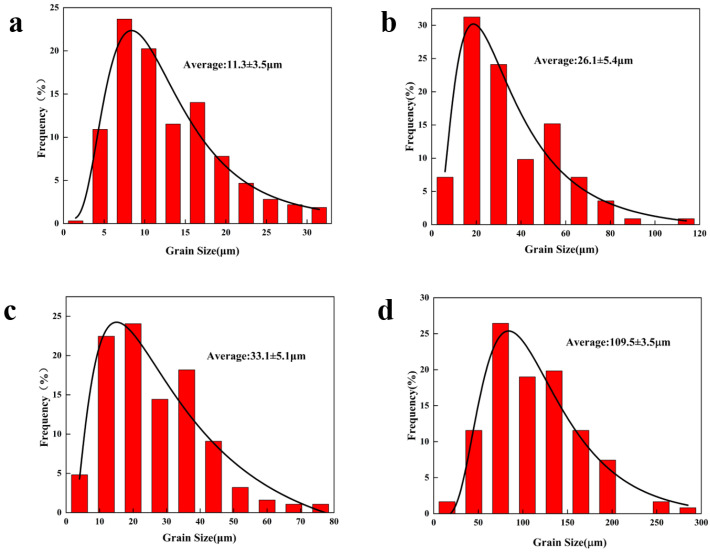
Grain size plots of the Mg-11.5Li-2.5Zn-0.35Al-0.3Y alloy at $\dot{\varepsilon }$ = 0.001 s^−1^ and *ε* = 0.6 with different temperatures: (**a**) 473 K, (**b**) 523 K, (**c**) 573 K, and (**d**) 623 K.

The microstructure and corresponding grain size of the Mg-11.5Li-2.5Zn-0.35Al-0.3Y alloy at different temperatures are shown in Figure 4 and Figure 5, respectively. It can be observed that the grain shape and size change significantly with the increase in temperature. Elongated deformed grains and equiaxed grains are presented in Figure 4a,b, and the elongated deformed grains decrease and the equiaxed grains increase with increasing temperature. This is mainly because of the fact that at a strain rate of 0.001 s^−1^, the driving force for the dynamic recrystallization of the deformed grains is more sufficient as the temperature increases, and the deformed grains gradually change from elongated to equiaxed. Equiaxed grains dominate in Figure 4c,d. The recrystallized grain size increases rapidly with increasing temperature, and the average grain size increases from 33.1 ± 5.1 μm Figure 5c to 109.5 ± 3.5 μm Figure 5d when the strain rate decreases from 0.01 s^−1^ to 0.001 s^−1^, an increase of about 3.3 times. Combined with Figure 4 and the subsequent hot processing diagrams, it is shown that the alloy is most suitably processed for plastic deformation at T = 473 K when $\dot{\varepsilon }$ = 0.001 s^−1^.

**Figure 6:**
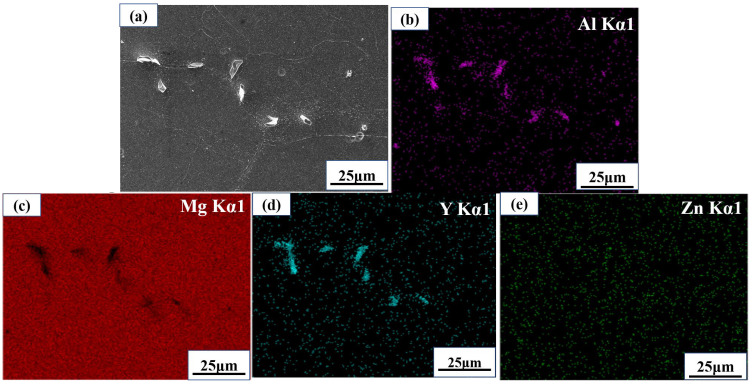
EDS images of the Mg-11.5Li-2.5Zn-0.35Al-0.3Y alloy under the T = 623 K, $\dot{\varepsilon }$ = 0.001 s^−1^, and *ε* = 0.6 deformation conditions: (**a**) SEM image; Elemental distribution: (**b**) Al, (**c**) Mg, (**d**) Y and (**e**) Zn.

Figure 6 shows the SEM micrograph and element distribution maps of the Mg-11.5Li-2.5Zn-0.35Al-0.3Y alloy. The massive secondary phase is mainly Al-rich- and Y-rich-dominated (Figure 6b,d), which suggests that the elements contained in this secondary phase are dominated by Al and Y. Further determinations of these phase compositions were accomplished using the EDS point-scanning process afterward. Zn exists in the Mg matrix as a form of solid solution; it is mainly uniformly distributed in the alloy (Figure 6e), and the aggregation of Al and Y did not occur. Therefore, it can also further indicate that the main secondary phase in this alloy does not appear to be a Zn-containing phase.

**Figure 7:**
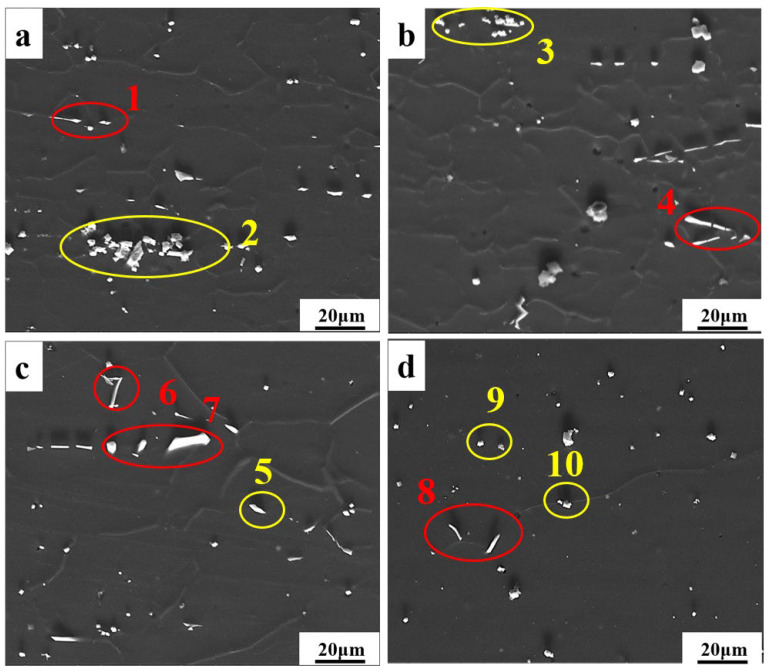
SEM maps of the Mg-11.5Li-2.5Zn-0.35Al-0.3Y alloy at T = 623 K and *ε* = 0.6 with different strain rates: (**a**) 1 s^−1^ (**b**) 0.1 s^−1^ (**c**) 0.01 s^−1^, and (**d**) 0.001 s^−1^.

**Figure 8:**
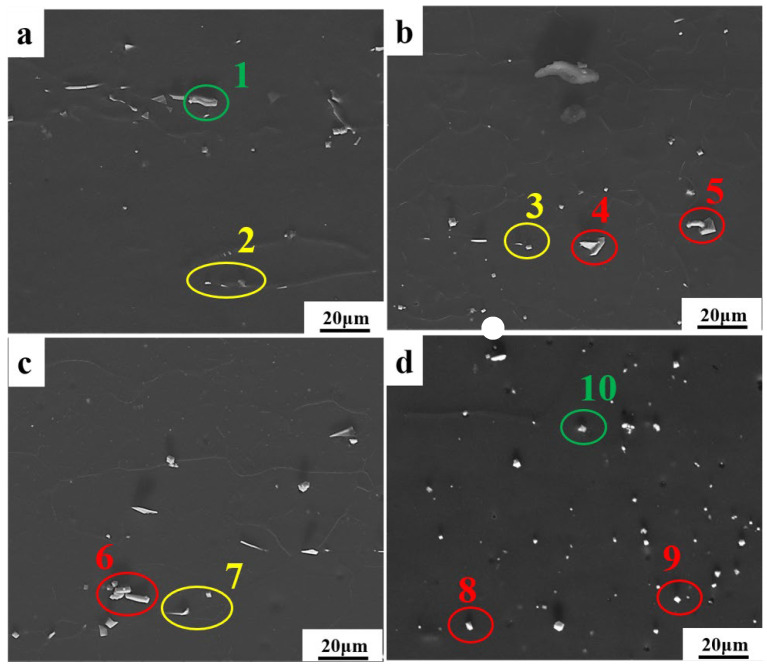
SEM images of the Mg-11.5Li-2.5Zn-0.35Al-0.3Y alloy at $\dot{\varepsilon }$ = 0.001 s^−1^ and *ε* = 0.6 with different temperatures at 0.001 s^−1^: (**a**) 473 K, (**b**) 523 K, (**c**) 573 K, and (**d**) 623 K.

The SEM images of the Mg-11.5Li-2.5Zn-0.35Al-0.3Y alloy at 623 K with different strain rates are shown in Figure 7. As can be seen from the images, the alloy consists of a darker β-Li matrix and a brighter Al_2_Y phase (labeled in red) and MgY phase (labeled in yellow), where the second phase is dominated by the Al_2_Y and MgY phases (Table 1). With the change in strain rate and temperature, Al_2_Y has always been a long strip with some changes in thickness, indicating that the shape of the Al_2_Y phase is less affected by the strain rate. The Al_2_Y phase is preferentially formed during the solidification process, and as a particle of non-uniform grain nucleation, it hinders grain growth and has the effect of refining grain and second-phase strengthening [19,20]. As the strain rate decreases, the MgY phase evolves from a larger block of an enriched state at a high strain rate to a dispersively distributed granular form at a low strain rate. Meanwhile, the temperature has less effect on the shape of the MgY phase. As shown in Figure 8, with the increase in temperature, the Al_2_Y phase gradually evolves from an elongated state to a granular state and is diffusely distributed in the β-Li matrix, while the MgY phase does not change as much (Table 2). We can find that the finer Al_2_Y phase and the dispersively distributed MgY phase contribute to the plastic deformation of the alloy, which is also consistent with the trend in the subsequent thermal processing maps.

### 3.2. Flow Behavior

The study of the deformation behavior of the Mg-11.5Li-2.5Zn-0.35Al-0.3Y alloy during hot compression requires considerations about the friction between the specimen and the mold [21,22,23], and the effect of friction was evaluated using the barrel coefficient (B), which is expressed as follows [24]:

(1) $$B=\frac{hR_{M}^{2}}{h_{0}R_{0}^{2}}$$

where *h*_0_ and *R*_0_ are the initial height and diameter of the specimen, respectively, and *h_M_* and *R_M_* are the height and maximum diameter of the specimen after thermal compression, respectively. When 1 < *B* ≤ 1.1, the effect of friction on the level of flow stress is small, and no correction of the stress–strain curve is needed; in contrast, when *B* > 1.1, it can be seen that the effect of friction on the level of flow stress is ignored, meaning that the stress–strain curve needs to be corrected. The barrel coefficients (Bs) of the specimens under each condition were evaluated, and as *B* ≤ 1.1, there was no need to correct the curves (Table 3).

**Figure 9:**
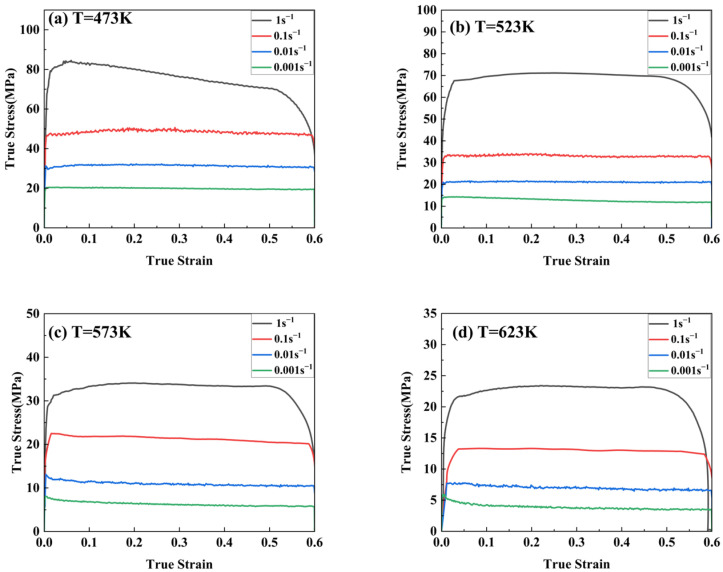
True stress-strain curves for Mg-11.5Li-2.5Zn-0.35Al-0.3Y at different temperatures and strain rates for a true strain of 0.6: (**a**) T = 473 K, (**b**) T = 523 K, (**c**) T = 573 K, and (**d**) T = 623 K.

When the deformation temperature is higher than 0.35~0.4 T_m_ (melting temperature), the level of external heat will be enough to affect the plastic deformation of the alloy, indicating that there is some correlation between the flow behavior, the deformation temperature, and the strain rate [25]. Figure 9 shows the stress–strain curves of the Mg-11.5Li-2.5Zn-0.35Al-0.3Y at different temperatures and strain rates for a strain variable of 0.6. From this figure, it can be concluded that the stress–strain curve exhibits dynamic reversion (DRV) and dynamic recrystallization (DRX) characteristics.

In all the curves, the level of stress increases with a decrease in temperature and an increase in strain rate. During the early stages of deformation, the flow stress increases dramatically with increasing strain, and the rate of increase gradually slows down until the peak stress is reached. Work hardening plays a dominant role in the low strain stage, which is a process of dislocation multiplication, entanglement, and proliferation. After reaching the peak stress, a part of the flow stress enters a stable stage (e.g., 473 K and 0.1 s^−1^) with the increase in strain, while another part of the flow stress decreases gradually with the increase in strain (e.g., 473 K and 1 s^−1^). The former is mainly the occurrence of continuous dynamic softening, and the latter is mainly the result of work hardening and continuous dynamic softening in an unbalanced state. In this stage, there are not only work hardening phenomena but also DRV and DRX phenomena [26]. In addition, with the work hardening and successive dynamic softening processes in dynamic balance, the stress-strain curve is serrated rather than smooth. It has been reported that this state of work hardening and dynamic softening of the alloys can be attributed to the interaction between solute atoms and dislocations [27].

**Figure 10:**
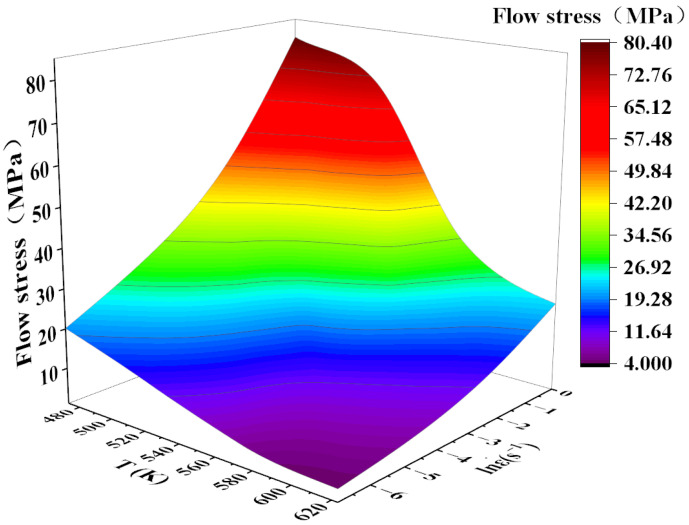
Stresses at different temperatures and strain rates for a true strain of 0.2.

Comparing the four graphs in Figure 9, the following conclusions can be drawn: Firstly, at the same temperature, the level of flow stress increases with an increase in strain rate. This may be due to the fact that the processing time decreases rapidly with the increase in strain rate, and although there is enough energy for dynamic recrystallization to occur, the degree of dynamic recrystallization that occurs in a very short period of time is very low, and a large amount of work-hardened structure (e.g., the long and stripy grains in Figure 2a) is retained. Meanwhile, the shorter the processing time, the finer the grain, which is consistent with the trends of Figure 2b–d. Secondly, at the same strain rate, the flow stress level decreases with the increasing in deformation temperature. This phenomenon indicates that the effect of work hardening weakened with increasing temperature, which can promote dislocation migration through the activation of the non-basal slip system, climbing, cross slip, and various DRX and DRV mechanisms (such as dislocation rearrangement and annihilation or grain boundary migration) at high temperatures, which ultimately promotes the formation of sub-granular grains and the recrystallization process, as shown in Figure 4a,b [28,29]. After the further increase in temperature, the recrystallized grains will grow larger, as shown in Figure 4c,d. Likewise, smaller values of flow stresses at low strain rates at high temperatures are also shown in Figure 10. This is mainly due to the lower dislocation stacking and propagation speeds at lower strain rates, which further weakens the effects of dislocation tangles and crossovers [30,31].

### 3.3. Constitutive Equation

The Arrhenius-type constitutive model was first proposed by Sellars and Tegart in 1996 and is nowadays commonly used to describe the thermal deformation behavior of Mg-Li alloys. It forms a link between flow stress, deformation temperature, and deformation speed. This model contains the deformation activation energy, *Q*, and the deformation temperature, *T*. It is usually expressed in one of the following three forms:

(1) In all stress states, the flow stress model is in the form of a hyperbolic sine function:
  (2) $${\dot{\varepsilon }=A[sinh(\alpha \sigma )]}^{n}exp(-Q/RT)$$
(2) At low stress levels, that is, when *σ* < 0.8, the flow stress model is in the form of a power exponential function:
  (3) $${\dot{\varepsilon }=A\sigma }^{n_{1}}exp[-Q/(RT)]$$
(3) In the high stress state, that is, when *σ* > 1.2, the flow stress model is in the form of an exponential function:

(4) $$\dot{\varepsilon }=Aexp(\beta \sigma )exp[-Q/(RT)]$$

where $\dot{\varepsilon }$ is the strain rate (s^−1^); *σ* is the peak stress in the stress-strain curve (MPa); *Q* is the deformation activation energy (J/mol); *R* is the gas constant (8.314 J/(mol·K)); *T* is the absolute temperature (K); and *A*, *α*, *β*, *n*_1_, and *n* are the material constants, where *A* is the structural factor and *n* is the stress index, and *α* is the stress level parameter *α* = *β*/*n*_1_.

Thermal plastic deformation of alloys is plastic deformation processing above the recrystallization temperature. In general, the temperature compensation factor Zener–Hollomon parameter (*Z* parameter) is used to characterize the effects of temperature and strain rate on the flow stress, *σ*, as follows:

(5) $$Z=\dot{\varepsilon }exp[Q/(RT)]$$

where *Z* is the temperature-compensated strain rate factor.

Combining the Zener–Hollomon parameters and the Arrhenius-type constitutive model in hyperbolic sine form, the expression of the flow stress, *σ*, with the *Z* parameter is as follows:

(6) $${{\sigma =\left(\frac{1}{\alpha }\right)ln\{(Z/A)}^{1/n}+[{\left(\frac{Z}{A}\right)}^{\frac{2}{n}}+1]}^{1/2}\}$$

Assuming that *Q* is not a function of *T*, Equations (7) and (8) were obtained through simultaneously taking the natural logarithms for both sides of Equations (3) and (4), respectively:

(7) $${ln\dot{\varepsilon }=n}_{1}ln\sigma +lnA-Q/(RT)$$

(8) $$ln\dot{\varepsilon }=\beta \sigma +lnA-Q/(RT)$$

**Figure 11:**
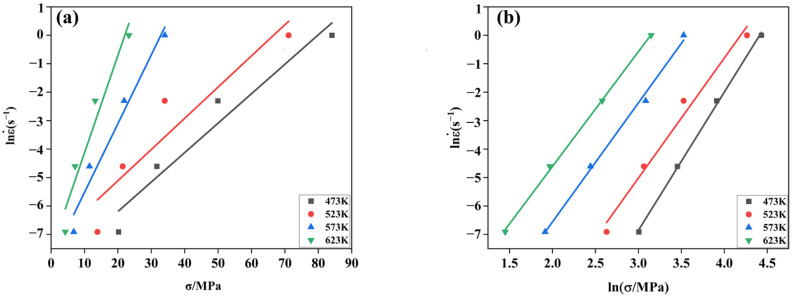
Linear correlation fitted curves: (**a**) *ln*$\dot{\varepsilon }\;$ − *lnσ*. (**b**) *ln*$\dot{\varepsilon }\;$ − *σ*.

It is easy to know from Equations (7) and (8) that *ln*$\dot{\varepsilon }$*-lnσ* and *ln*$\dot{\varepsilon }$−*σ* are linearly related with slopes *n*_1_ *=* [∂*ln*$\dot{\varepsilon }$*/*∂*lnσ*]*_T_* and *β* = [∂*ln*$\dot{\varepsilon }$/∂*σ*]*_T_*, respectively. Taking (*lnσ*, *ln*$\dot{\varepsilon }$) and (*σ*, *ln*$\dot{\varepsilon }$) as the coordinate points, linear regression analysis was performed using OriginPro 2021 (Learning version) processing software; four fitted curves were obtained, as shown in Figure 11, and the slopes of the four fitting curves were averaged to obtain *n*_1_ = 4.3217 ± 0.4623 and *β* = 0.1993 ± 0.0047. Subsequently, it was calculated that *α* = *β/n*_1_ = 0.0461 ± 0.0102. It can be seen from Figure 11 that both *ln*$\dot{\varepsilon }$ − *lnσ* and *ln*$\dot{\varepsilon }\;$− *σ* exhibit linear relationships with high correlation strengths, indicating that the flow stresses and strain rates satisfy both the low-stress low function relationship of the Arrhenius-type constitutive model and the high-stress exponential function relationship.

**Figure 12:**
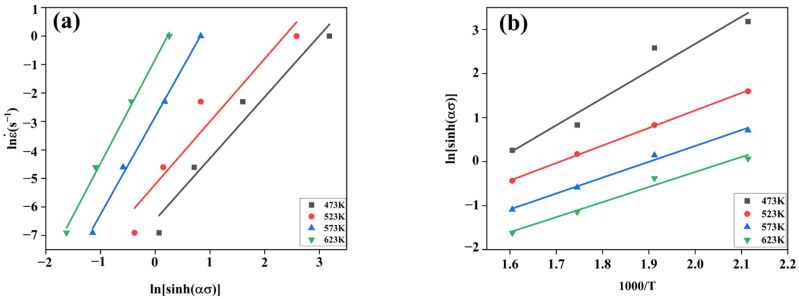
Linear correlation fitted curves: (**a**) *ln*$\dot{\varepsilon \;}$ − [*sinh*(*ασ*)]. (**b**) [*sinh*(*ασ*)] − 1000*/T*.

Similarly, taking natural logarithms on both sides of Equation (2) at the same time led us to obtain Equation (9), from which *ln*$\dot{\varepsilon }\;$− [*sinh*(*ασ*)] was found to be linear with slope *n* = {*∂ln*$\dot{\varepsilon }$*/∂ln*[*sinh*(*ασ*)]}. Linear regression analysis was performed with (*ln*$\dot{\varepsilon }$, *ln*[*sinh*(*ασ*)]) as the coordinate point, and the graphs of the fitted relationship are shown in Figure 12a. Taking the average of the slopes of the four fitted straight lines, *n* = 2.8600 ± 0.2704 was obtained.

(9) $$ln\dot{\varepsilon }=nln\left[\sinh\left(\alpha \sigma \right)\right]+lnA-\frac{Q}{RT}$$

The deformation and derivation of Equation (9) led to the formation of Equation (10):

(10) $$Q=R{\left\{\frac{\partial In\dot{\varepsilon }}{\partial ln\left[\sinh\left(\alpha \sigma \right)\right]}\right\}}_{T}{\left\{\frac{\partial ln\left[\sinh\left(\alpha \sigma \right)\right]}{\partial \left(\frac{1000}{T}\right)}\right\}}_{\dot{\varepsilon }}=RnK$$

where *K* is the average slope of the curve fitted to the linear relationship of *ln*[*sin*(*ασ*)] −1000/*T* (Figure 12b), with *K* = 4292.5700 ± 0.4269. The deformation activation energy *Q* = 102.0701 ± 0.9597 (KJ/mol) was obtained by taking the material constants obtained above into Equation (10).

Taking the natural logarithm on both sides of Equation (5) led us to develop Equation (11):

(11) $$lnZ=lnA+nln\left[\sinh\left(\alpha \sigma \right)\right]$$

**Figure 13:**
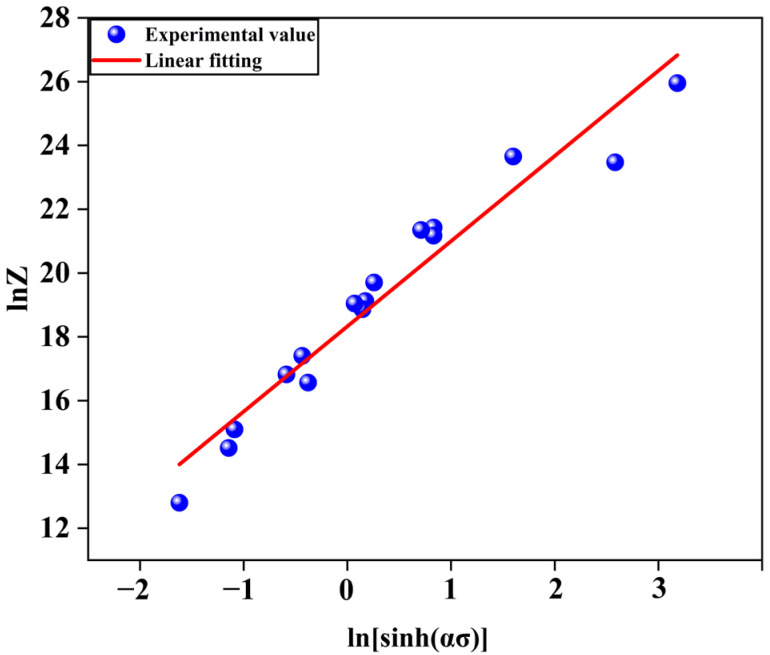
*lnZ* − *ln*[*sinh*(*ασ*)] linear relationship.

From Equation (11), it can be seen that *lnZ* − *ln*[*sinh*(*ασ*)] is a linear relationship, and the linear data are fitted with (*ln*[*sinh*(*ασ*)], *lnZ*) as the coordinate point, and the fitted linear plot is shown in Figure 13, and *lnZ* is the intercept of the fitting curve, which retrieves *lnA* = 18.3305 ± 0.2286 and *A* = 9.1374 × 10^7^ ± 1.2569 through calculation.

It can be seen from Figure 13 that the linear regression correlation coefficient *R* = 0.9690 ± 0.2286 indicates that the relationship between the flow stress and the deformation temperature and strain rate of the multicomponent Mg-11.5Li-2.5Zn-0.35Al-0.3Y alloy during the hot compression experiment can be described using the hyperbolic sine function in the Arrhenius-type constitutive model.

The calculated related material constants are shown in Table 4.

Substituting the data from the table into Equation (2), we can obtain the constitutive equation for the multicomponent Mg-11.5Li-2.5Zn-0.35Al-0.3Y experimental alloy:

(12) $$\dot{\varepsilon }={{9.1374\times 10}^{7}[\sinh\left(0.0461\sigma \right)]}^{2.8600}exp(-\frac{102070.1}{RT})$$

### 3.4. Characterization of Processing Maps

Gegel and Prasad et al. [32,33] proposed the DMM theory. Based on the DMM theory, the dynamic material model can be used to draw the processing map, which can reflect the relationship between the flow behavior, hot processing properties, and deformation parameters of metal materials during hot processing. By analyzing the hot processing map, it is possible to predict the deformation mechanism of the material at different deformation temperatures and deformation rates, and it can also obtain the stable and unstable regions of hot processing, which can help to control the evolution of the microstructure in the future, avoid machining defects, and optimize the processing technique. According to the DMM model, the total energy absorbed by the workpiece (*P*) consists of two complementary functions: *G* represents the dissipated energy by the material during thermoplastic deformation, and *J* represents the energy consumed by the material during microstructural changes.

(13) $$P=\sigma *\dot{\varepsilon }=G+J=\int_{0}^{\dot{\varepsilon }}\sigma d\varepsilon +\int_{0}^{\sigma }\int_{0}^{\dot{\varepsilon }}\varepsilon d\sigma$$

The strain rate sensitivity constant, *m*, represents the proportion of *G* and *J* energies at a given deformation temperature and strain.

(14) $$\frac{dJ}{dG}=\frac{\dot{\varepsilon }d\sigma }{\sigma d\dot{\varepsilon }}=\frac{d\left(ln\sigma \right)}{d\left(ln\dot{\varepsilon }\right)}\approx \frac{∆log\sigma }{∆log\dot{\varepsilon }}=m$$

The efficiency of power dissipation (*η*) was used to describe the proportion of total energy consumed by microstructural changes in a material during the hot processing procedure, as follows:

(15) $$\eta =\frac{2m}{m+1}$$

Based on the DMM theory, through mathematical transformations, the Prasad instability criterion can be expressed as:

(16) $$\xi \left(\dot{\varepsilon }\right)=\frac{\partial ln\frac{m}{m+1}}{\partial ln\dot{\varepsilon }}+m<0$$

where the *ξ* ($\dot{\varepsilon }$) instability factor is a dimensionless constant; the value of *ξ* ($\dot{\varepsilon }$) at different deformation temperatures, strain rates, and strain can be used to draw the instability map of the material, and the hot processing map can be obtained by superimposing the instability map on the power consumption map. In the hot processing map, the region where *ξ* ($\dot{\varepsilon }$) < 0 indicates the instability region of the material during thermoplastic deformation, and since the material is prone to flow instability in the instability region, the instability region needs to be avoided during thermoplastic deformation. Many researchers have summarized the causes of flow instability as flow localization, adiabatic shear bands, dynamic strain aging, kink bands, and flow rotating lights of deformation twins [33,34,35]. *ξ* ($\dot{\varepsilon }$) > 0 indicates the stable region of a material during thermoplastic deformation, where the material undergoes a complete recrystallization in the process of thermoplastic deformation, which results in the elimination of internal stresses, a substantial reduction in dislocations and deformation microstructure, and improved hot processing properties of the material.

**Figure 14:**
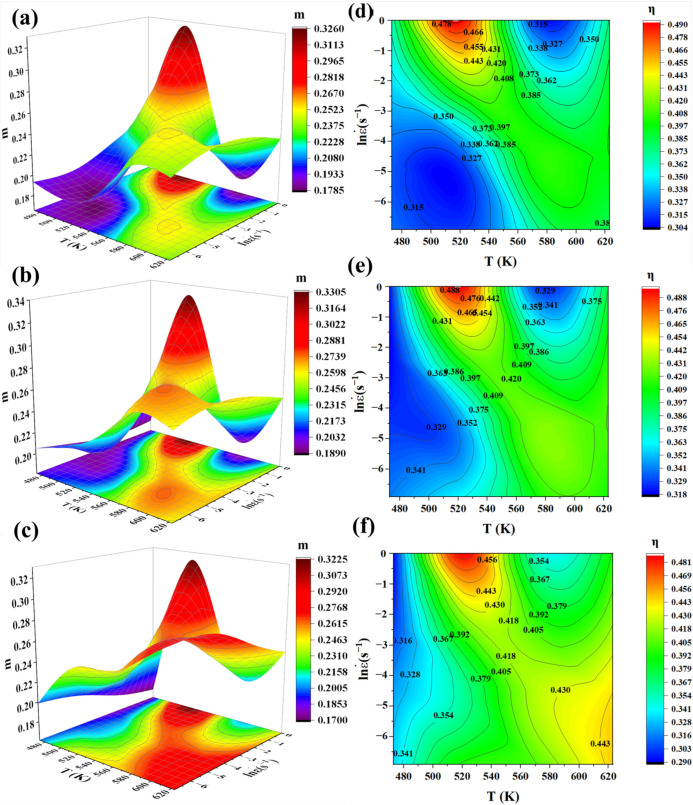
Three-dimensional plots of strain-sensitive indices (m) and hot processing maps: (**a**,**d**) *ε* = 0.1; (**b**,**e**) *ε* = 0.3; and (**c**,**f**) *ε* = 0.5.

From Figure 14a–c, it can be seen that when the temperature is between 473 K and 623 K, the strain rate is between 1 s^−1^ and 0.001 s^−1^, and the strain is 0.1, 0.3, and 0.5, respectively; the change trend of the strain rate sensitivity index, *m*, is basically the same. The value of m can reflect the general trend of the change in energy consumption in the process of microstructural transformation, and it can also reflect the plasticity of the material under certain conditions. When the deformation temperature is between 550 K and 623 K, the value of *m* decreases with the increase in strain rate, and the plasticity of the material decreases. This is consistent with Figure 2a: when the deformation temperature is high but the strain rate is large, the microstructure contains some deformed microstructure, and the existence of such a deformed microstructure will reduce the plasticity of the material. Additionally, no matter whether the strain is 0.1, 0.3, or 0.5, the trend of the value of *m* is always between 0.1700 and 0.3305, which indicates that the influence of the strain has little effect on the value of *m*, and it is more the influence of the deformation temperature and strain rate that is larger.

The hot processing maps with strains of 0.1, 0.3, and 0.5 are shown in Figure 14d–f, respectively, where the numbers on the contour lines indicate the energy dissipation coefficient, *η*, and the value of *η* can reflect the dynamic energy dissipation capacity of the alloy. The peak value of *η* is located in the region where the strain rate is between 0.368 s^−1^ and 1 s^−1^, and the deformation temperature is between 500 K and 540 K at the peak value of *η* in the three variables, which is about 48%. The minimum value of *η* is located in both the low temperature and low strain rate or the high temperature and high strain rate of the maps with strains of 0.1 and 0.3, which is about 32%, while it is only located in the low temperature of the map with a strain of 0.5, which is about 32%. The values of *η* reflect the dynamic energy consumption ability of the alloy. There is no plastic deformation instability region in the hot processing map, which indicates that the alloy can safely undergo a safe hot plastic deformation at temperatures between 473 K and 623 K, both at strain rates between 0.001 s^−1^ and 1 s^−1^. Combined with the microstructure of the alloy (e.g., Figure 2 and Figure 4), no defects, such as cracks, were found. 

## 4. Conclusions

The hot deformation behavior of the Mg-11.5Li-2.5Zn-0.35Al-0.3Y alloy was systematically investigated using the constitutive model and hot processing maps at temperatures ranging from 473 K to 623 K and strain rates in the range from 0.001 s^−1^ to 1 s^−1^. The microstructural evolution of the alloy was analyzed using metallography (OM), scanning electron microscopy (SEM), and energy-dispersive spectroscopy (EDS). The following conclusions can be drawn based on the experimental results:

(1) Regarding the Mg-11.5Li-2.5Zn-0.35Al-0.3Y alloy, at a temperature of 623 K, its rheological stresses appear to increase rapidly in the grains at a strain rate of 0.01 s^−1^~0.1 s^−1^. At a strain rate of 0.001 s^−1^ and temperatures ranging from 573 K to 623 K, the grains appear to increase rapidly. This alloy should be processed under these conditions when thermoplastic processing is avoided as much as possible.
(2) By calculating the deformation activation energy of the Mg-11.5Li-2.5Zn-0.35Al-0.3Y alloy, *Q* = 102.0701 (KJ/mol), the peak stress of this alloy can be described using the following constitutive equation:
  $$\dot{\varepsilon }={{9.1374\times 10}^{7}\left[\sinh\left(0.0461\sigma \right)\right)}^{2.8600}exp[-\frac{102070.1}{RT})]$$
(3) The accuracy of the model was verified by comparing the model’s prediction values with the experimental values. The correlation coefficient R = 0.9690 was calculated, indicating that the established stress-compensated Arrhenius-type constitutive model is able to well describe the relationship between the flow stress and the deformation temperature and strain rate of the Mg-11.5Li-2.5Zn-0.35Al-0.3Y alloy during hot compression.
(4) Based on the DMM theory and instability criterion, the hot processing maps under different strains were established, and the Mg-11.5Li-2.5Zn-0.35Al-0.3Y alloy can safely undergo hot compression and plastic deformation between the strain rates of 0.001 s^−1^ and 1 s^−1^ and temperatures of 473 K~623 K. To improve the properties of this alloy, plastic deformation processing can be performed at low temperatures and high strain rates.

## Figures and Tables

**Table 1:** Elemental contents of the points in Figure 7 (At%).

Different Specimen	Position	Mg	Zn	Al	Y
a	1	53.7	6.1	25.7	14.4
	2	48.2	0.0	7.9	43.9
b	3	48.2	1.5	2.4	47.9
	4	49.3	5.2	30.0	15.5
c	5	21.7	7.7	43.6	26.9
	6	68.7	3.3	18.8	9.2
	7	53.0	5.2	27.3	14.5
d	8	42.7	6.7	33.1	17.5
	9	46.8	0.0	4.2	49.0
	10	50.9	0.0	4.0	45.1

**Table 2:** Elemental contents of the points in Figure 8 (At%).

Different Specimen	Position	Mg	Zn	Al	Y
a	1	58.2	4.1	24.9	12.7
	2	35.4	0.0	17.3	39.4
b	3	39.6	0.0	15.0	45.4
	4	22.3	9.1	46.6	22.0
	5	18.5	0.0	54.3	27.2
c	6	9.7	0.0	58.6	31.7
	7	42.9	0.0	13.6	43.5
d	8	42.6	0.0	37.7	19.7
	9	40.8	2.1	40.8	20.6
	10	81.8	0.0	2.5	15.7

**Table 3:** Barrel coefficients (Bs) of the Mg-11.5Li-2.5Zn-0.35Al-0.3Y alloy at different temperatures and strain rates.

	473 K	523 K	573 K	623 K
1 s^−1^	1.0685	1.0870	1.0632	1.0451
0.1 s^−1^	1.1067	1.0567	1.0567	1.0169
0.01 s^−1^	1.0578	1.0505	1.0505	1.0357
0.001 s^−1^	1.0467	1.0293	1.5627	1.0504

**Table 4:** Material constants of Mg-11.5Li-2.5Zn-0.35Al-0.3Y alloys.

*α*	*n*	*A*	*Q* (kJ/mol)
0.0461 ± 0.0102	2.8600 ± 0.2704	9.1374 × 10^7^ ± 1.2569	102.0701 ± 0.9597

where *A* is the structural factor; *Q* is the deformation activation energy; *n* is the stress index; and *α* is the stress level parameter *α* = *β/n*_1_.

## Data Availability

Data are contained within the article.
